# Contribution of Major Groups of Food Products to the Daily Intake of Selected Elements—Results from Analytical Determinations Supported by Chemometric Analysis

**DOI:** 10.3390/nu12113412

**Published:** 2020-11-06

**Authors:** Wojciech Koch, Marcin Czop, Agnieszka Nawrocka, Dariusz Wiącek

**Affiliations:** 1 Department of Food and Nutrition, Medical University of Lublin, 4a Chodźki Str., 20-093 Lublin, Poland; 2 Department of Clinical Genetics, Medical University of Lublin, Radziwiłłowska 11 Str., 20-080 Lublin, Poland; marcin.czop@umlub.pl; 3 Institute of Agrophysics, Polish Academy of Sciences, Doświadczalna 4 Str., 20-290 Lublin, Poland; a.nawrocka@ipan.lublin.pl (A.N.); d.wiacek@ipan.lublin.pl (D.W.)

**Keywords:** food, dietary intake, elements, F-AAS, ICP-OES

## Abstract

Food is a major source of minerals for humans. The main objective of this study was to determine the intake level of 10 essential macro- (Na, K, Ca, and Mg) and trace elements (Cu, Zn, Mn, Fe, Cr, and Se) with major food groups among young adults. Dietary intake of elements was evaluated using the 24-h dietary recall technique in combination with F-AAS and ICP-OES methods. A very high intake of sodium and a very low intake of calcium, combined with inappropriate sodium/potassium ratio, may be harmful to the health of the population. Dietary intake of trace elements was within the range of reference values in the subjects, with cereals being the major source of a majority of those elements, while meat (38% for Na), vegetables (25% for K), and milk products (75% for Ca) were the main contributors to the daily dietary intake of macroelements. PCA revealed several visible trends in the datasetAmong men, the intake of Zn, Cr Na and K was significantly correlated with the consumption of meat and vegetables, whereas Mg, Se, Fe and Cu with cereals and water and beverages. Among women, the intake of Mg was significantly correlated with the consumption of meat and vegetables.

## 1. Introduction

Food is an integral part of human existence. Every day, along with the food, humans receive a certain amount of nutrients, most importantly mineral substances, which determine their health, development, and physical condition [1]. Except for a small group, such as workers exposed to some toxic elements via inhalation or dermal contact at the workplace, food consumption has been the main route of intake of all essential elements for the general population [2,3]. Considering the intake level, elements can be classified into macro- and trace elements, while based on their chemical properties, they can be classified into metals (e.g., sodium, potassium, calcium, or iron) and non-metals (selenium or iodine). In addition to essential elements, foods may also be a source of toxic elements, which do not play any positive role in humans’ health and have a negative impact. In general, they are called “heavy metals”—a term referring to a group of metals (cadmium, mercury, lead, or nickel) and metalloids (arsenic) [4,5].

Studies on the concentration of various elements present in the food originating from plants and animals have been conducted for many decades, which may aid in determining the main dietary sources of essential as well as toxic elements for a general or specific population. A very large number of publications on the intake of selected minerals, mainly metals, appear every year in the global scientific literature [3,6,7,8,9,10,11]. Such studies not only compare the consumption of individual substances with the recommended values but also compare the eating trends between different parts of the world and track their variability in different regions or countries. Moreover, the results thus obtained contribute to correcting eating habits and thus are of great importance in the prevention of diet-related diseases and for medical and food services in particular countries [12].

The main objective of this study was to determine the intake level of 10 essential macro- (Na, K, Ca, and Mg) and trace elements (Cu, Zn, Mn, Fe, Cr, and Se) with major food groups among young adults. The results obtained were subjected to chemometric analysis to statistically determine the most important foods that significantly contribute to the total intake of particular elements. To reduce the number of variables and to detect the structure of relationships between variables, principal component analysis (PCA) was used. It was assumed that the intake of the tested elements would be inappropriate in some cases. Thus, additionally, the study aimed to assess and compare the daily intake of the determined minerals with appropriate reference values and worldwide trends. Adequate evaluation of food intake is important for determining the dietary trends and assessing the effects of interventions on the population. However, the determination of dietary intake is challenging since food consumption is a complex phenomenon that is very difficult to analyze; thus, a suitable food intake methodology is required in order to obtain reliable data [12]. Studies on mineral intake, which are based on analytical determinations, are difficult to conduct and more expensive; thus, the majority of the data found in the scientific literature are based on computer evaluation. However, data obtained from analytical determinations better indicate the actual nutrient intake in a specific population [8,9,10,11]. This study used a combination of various methods required to obtain reliable nutritional data. Dietary intake data were assessed using the 24-h dietary recall technique, which is the best self-report dietary assessment instrument [12,13,14]. The resulting data were processed using Dieta 5.0 Software (National Food and Nutrition Institute, Warsaw, Poland), which is recognized as the most suitable software for processing nutritional data by the Polish Food and Nutrition Institute in Warsaw. Based on the processed data, diet samples were reconstructed by determining the concentrations of various elements using flame atomic absorption spectrometry (F-AAS) and inductively coupled plasma optical emission spectrometry (ICP-OES). Subsequently, the gathered data were subjected to statistical analysis.

## 2. Materials and Methods

### 2.1. Chemicals

All reagents used in the study were of Suprapur grade and purchased from Darmstadt (Germany). High-purity deionized water (resistivity 18.2 M Ωcm) obtained from a Milli-Q water purification system (Millipore, Bedford, MA, USA) was used throughout the study. Teflon vials and polypropylene recipients were used for sample digestion and the storage of digests, respectively.

### 2.2. Sampling

The study was conducted from October 2016 to March 2018. Data on food intake were obtained using 24-h dietary recall technique based on the author’s questionnaire. Only midweek days were reported. The study involved 579 healthy participants (274 women and 305 men), who were students of the Medical University of Lublin, residing in the eastern part of Poland (Lublin and its province). The study was anonymous, and all the participants were volunteers. Pregnant and lactating mothers were excluded from the study. The level of physical activity was checked using additional questions. Table 1 characterizes the studied population. Before data collection, oral informed consent was obtained from all participants by the data collectors. The Institutional Review Board of the Division of Pharmacy, Medical University of Lublin, approved all the study procedures and materials. Before the study, participants were informed of the purpose of the study and that all the information is anonymous. Participants were interviewed in the Department of Food and Nutrition of the Medical University of Lublin. All the collected questionnaires were checked for correctness in terms of their completion before they were used in the study. The collected data were processed by using two programs—Dieta 5.0 Software (National Food and Nutrition Institute, Warsaw, Poland) and Dietetyk 2006 (Jumar, Poland)—to average all the food rations for women and men, considering the classification of the main food products. According to a guideline of the National Institute of Food and Nutrition in Warsaw, the intake of elements was analyzed with the main food products classified into 12 groups.

The average main food groups were prepared in duplicates based on computer-processed data. All the products used to prepare partial food rations were from the retail market of the Lublin region. Since this is an agricultural region, over 90% of the most important products—cereals, milk and its products, meat and its products, eggs, vegetables, and fruits—were locally sourced. For each food group, three separate duplicates were prepared. In terms of the gender, number of food groups, reconstructions and repetitions (2 × 12 × 3 × 3), a total of 216 food samples were subjected to analytical determinations.

The preparation of the food items in the laboratory was undertaken in ways similar to the local cooking practices. The book of Nadolna and coworkers was referred to for preparing various meals and dishes [15]. As per the international WHO/GEMS (World Health Organization/Global Environment Monitoring System) food recommendations, dishes were prepared using drinking water from the city where the foods were bought; in this study—Lublin [3,16]. Salt and spices were added to the foods similar to regular cooking practices. The non-edible parts (bones, fish bones) were removed. Particular food items were homogenized using titanium blades and stored in plastic containers at −20 °C before the analysis. Cooking utensils made of aluminum, ceramic, or enamel were avoided during the preparation of food products or meals. Before food preparation, the equipment used for preparing and homogenizing the composite samples was thoroughly washed with a laboratory-grade detergent, and rinsed well with hot tap water, 20% nitric(V) acid of suprapur grade, and finally with deionized water to avoid cross-contamination.

### 2.3. Digestion of Samples

The homogenized food samples (40 ± 0.01 g) were weighed into quartz crucibles and digested following a previously described protocol [16,17]. Briefly, the samples were initially dried at 105 °C in an electric dryer and ashed at 450 °C in a muffle furnace for 72 h. This process was accelerated using a 30% water solution of nitric (V) acid, which was then evaporated and the samples were reheated at 250 °C. The resulting residue was dissolved in 10% hydrochloric acid and diluted with deionized water (1 + 1, *v/v*). The digests thus obtained were transferred quantitatively into separate test tubes, filtrated through a filter paper to remove silica and other insoluble particles, and made up to a volume of 25 mL with deionized water. Simultaneously, six samples of reference material (10 g each) were weighed in quartz crucibles and subjected to the protocol described above (these did not require drying).

### 2.4. Analytical Determination of Elements by Flame Atomic Absorption Spectrometry (F-AAS) and Inductively Coupled Plasma Optical Emission Spectrometry (ICP-OES)

The amount of Na, K, Mg, Ca, Cu, Zn, Mn, and Fe was determined directly from the digests with an F-AAS SOLAAR M5 apparatus (Thermo Scientific, Waltham, MA, USA) using, if required, 10-, 1000-, and 5000-fold dilutions. Lanthanum trichloride was added as a spectral buffer at a final concentration of 10% to the analyzed samples during calcium determinations.

The amount of Cr and Se was determined using the ICP-OES method with an iCAP 6500 Duo, inductively coupled plasma optical emission spectrometer (Thermo Fisher Scientific, Waltham, MA, USA), which was controlled by PC-based iTEVA software. The following instrumental settings were used: RF generator power of 1150 W, RF generator frequency of 27.12 MHz, coolant gas flow rate of 16 L·min^−1^, carrier gas flow rate of 0.65 L·min^−1^, auxiliary gas flow rate of 0.4 L·min^−1^, maximum integration time of 15 s, pump rate of 50 rpm, axial viewing configuration flush time of 20 s; three replicates were analyzed. Multielement standards CCS-4 and CCS-6 (100 µg/mL in 7% HNO_3_; Inorganic Ventures, Christiansburg, VA, USA) were used for calibrating the concentration of elements.

Although the method was previously validated [17], this study checked for its accuracy in the analysis of selected elements in food samples. For this, a mixture of flour and milk powder (in the ratio 70:30), fortified with known concentrations of investigated elements, was used as reference material. The results obtained from the accuracy analysis are shown in Table 2. Analysis of both the samples and reference material was performed simultaneously under the same conditions.

### 2.5. Statistical Analysis

Statistical analysis was performed using Microsoft Excel 2010, Statistica 13.3 (StatSoft, Kraków, Poland) and Graph Pad Prism 7 (Graph Pad Software, San Diego, CA, USA). Data were analyzed for normal distribution using the Shapiro–Wilk test. The statistical significance of macroelements and trace elements between particular food groups and sex were analyzed using two-way analysis of variance (ANOVA, with two qualitative factors) followed by Tukey’s test. To reduce the number of variables and to detect the structure of relationships between variables, principal component analysis (PCA) was used. The selection of the number of components was carried out in accordance with the Kaiser criterion and the scree plot. All results are presented as mean ± standard deviation (SD). The level of statistical significance was set at *p* ≤ 0.05.

## 3. Results

### 3.1. Distribution of Food Groups

Food intake among women and men is presented in Table 3.

Data presented in Table 3 revealed that, among solid foods, vegetables were consumed in the highest amounts, among both women and men. Other important food groups consumed by women were milk and dairy, fruits, cereals, and meat and meat products. Among men, vegetables were mainly consumed followed by cereals, meat, and dairy products. Apparently, both the genders consumed a low amount of fish. This distribution of food groups is different compared to other studies conducted in Poland, which indicated that cereals were consumed in the highest amounts. Moreover, the present study revealed that the consumption of meat and sweets and sugars was also much higher compared to that shown by previous research performed among an adult Polish population [3]. In another study, Sousa and Da Costa [12] revealed that the Brazilian adult population consumed fruits and cereals in the highest amounts and indicated a very high consumption of meat and sugars, which is in agreement with the results of the present study.

### 3.2. Dietary Intake of Macroelements with Major Food Groups

The contribution of major food groups to the total daily intake of sodium, potassium, magnesium, and calcium among women and men is presented in Table 4. Daily intake of particular elements was compared with the actual Polish dietary reference intake standards defined by the National Institute of Food and Nutrition [18], which are mandatory for the adult population in Poland. The daily sodium intake was compared to the value of adequate intake (AI), and the intake of three other macroelements was compared to the estimated average requirement (EAR), which is considered the most appropriate to compare the average intake level in the population [12,18,19,20,21,22].

Obtained results showed that meat and meat products contribute to almost 38% of sodium intake among men and 32% among women. Another highly processed food group—cereals—accounted for 28% and 24% of the total daily sodium intake among women and men, respectively. Importantly, these two groups of products accounted for over 60% of the daily sodium intake.

Daily potassium intake, in both genders, was much below the recommended AI value. The results of the present study shed new light on the contribution of various food products to the total potassium intake and reveal the dietary differences between the genders. Among women, vegetables were the main food group contributing to 25% of the total potassium intake, followed by fruits (18.1%), meat and meat products (17.9%), cereals, and milk and dairy (both around 11.3%). On the other hand, among men, the main contributors to potassium intake were meat and meat products (28%), followed by vegetables (19%), fruits (11.4%), and cereals (10.6%).

Dietary intake of magnesium was found to be below the recommended EAR values; however, significant differences were observed between genders in terms of fulfillment of the reference values. Although among women the mean intake reached almost 90% of the EAR, among men it was below 70% of this value. Cereals were identified to be the major contributors to the overall magnesium intake, accounting for 28.5% and 31.8% of the total daily intake of magnesium, among women and men, respectively. Meat and meat products were the second major food group significantly contributing to magnesium intake (16.9% and 17.4%), followed by vegetables (15.3% and 10.2%), fruits (15.3% and 8.2%), and milk and dairy products (10% and 9.6%), among women and men, respectively.

Dietary intake of calcium was found to be very low in the studied population—below 50% of the EAR value among women and only 54% of the recommended intake among men. In the present study 73.8% and 75.1% of daily calcium intake, among women and men, respectively, was derived from milk and dairy products. The contribution of other food groups to the total calcium intake was significantly lower—vegetables being the second most important group (11.2% and 7.2%), followed by eggs (4.9% among men) and cereals (3% and 3.2%) among women and men, respectively.

### 3.3. Dietary Intake of Trace Elements with Major Food Groups

The contribution of major food groups to the total daily intake of copper, zinc, manganese, iron, chromium, and selenium, among women and men, respectively, is presented in Table 5. 

Daily copper intake was almost equal among women and men. Cereals were identified as the most important products, accounting for 33% of the daily copper intake, followed by vegetables (17% and 14.4%) and meat and meat products (13.2% and 13.7%), among women and men, respectively. Interestingly, fruits were found to be a significant contributor to the overall copper intake and provided 14.3% of the daily intake among women and 11.3% among men.

Zinc consumption contributed to 129% and 109% of the EAR value, among women and men, respectively. The results of this study revealed that meat and meat products contribute the most to overall zinc intake, accounting for 28.1% and 33.2% of the daily intake among women and men, respectively. Other important food groups that significantly contribute to total zinc intake were cereals (24.6% among women and 22.7% among men) and milk and dairy products (22% among both women and men). The contribution of vegetables to overall zinc intake was much lower, yet much higher among women (10.1%) compared to men (6.9%).

The mean intake of manganese was more than twice the recommended AI values, in both women and men. The results of the current investigation revealed that cereal products were the major contributors to overall manganese intake among women, accounting for 39.5% of the daily intake, followed by water and beverages (24.7%), fruits (12.9%), and vegetables (11.1%). However, the results obtained among men were slightly different—water and beverages accounted for 53.5% of the daily manganese intake, followed by cereals (27.7%) and vegetables (6.77%). Since Mn concentration in water from the Lublin region is very low (<10 µg/L), it is clear that high manganese intake from this food group is mainly related to the consumption of tea. The daily intake of iron was above the reference values, among both women and men. Cereals were found to be the major contributor to the overall iron intake, accounting for 40.9% and 43.7% of the daily intake, followed by meat and meat products (15.7 and 13.8%) and vegetables (11.3 and 12%), among women and men, respectively. Moreover, the study revealed that the significant contributors to the general iron intake were sweet and sugars (accounting for 12.1 and 10.1% of iron intake, among women and men, respectively). The contribution of other food groups (e.g., milk and dairy, fruits, or eggs) to the overall iron intake, among both women and men, was below 5% and was therefore considered insignificant.

The results obtained in the current investigation confirmed that the intake level of chromium is far above the recommended AI value, both in women and in men. The level of intake from particular food groups differed significantly between the sexes. Cereals were the main contributors, accounting for 23.2% and 23.4% of the daily intake of chromium, among women and men, respectively. Among women, the second most important food group significantly contributing to the overall Cr intake was sweets and sugars (21.5%), followed by meat and meat products (16.1%) and milk and dairy (20.3%). Among men, the second most important food group was meat and meat products (22.9%), followed by milk and dairy (20.3%) and vegetables (9.66%). Among men, the contribution of sweets and sugars to the overall chromium intake was also significant (9.2%); however, it was much lower compared to women. Interestingly, daily chromium intake from three major groups of food (cereals, meat, and milk and dairy) among men accounted for almost 70% of the total intake of this trace element.

The daily intake of selenium was greatly above the recommended reference values. The results of the present study show that the structure of selenium intake significantly differs between the genders. Among women, cereals were the main contributors (28.2% of the daily intake), followed by water and beverages (20.9%), vegetables (14.5%), and fruits (10.2%). Surprisingly, in contrast to the results obtained in other countries, the contribution of foods such as meat (6.3%), milk and dairy products (4.7%), fish (1.4%), or eggs (<1%) to the overall selenium consumption was very low. Among men, the distribution of selenium intake was different, with cereals being the most important contributor (28.2%), followed by vegetables (18.8%), meat (14.5%), and fruits (10.2%). However, the contribution of water and beverages was significant, accounting for almost 10% of the daily selenium intake. On the other hand, similar to the results obtained among women, the contribution of food groups such as milk and dairy (7.7%), fish (1.8%), and eggs (<1%) to the total selenium intake was very low.

### 3.4. Principal Component Analysis (PCA) Analysis

**Figure 1:**
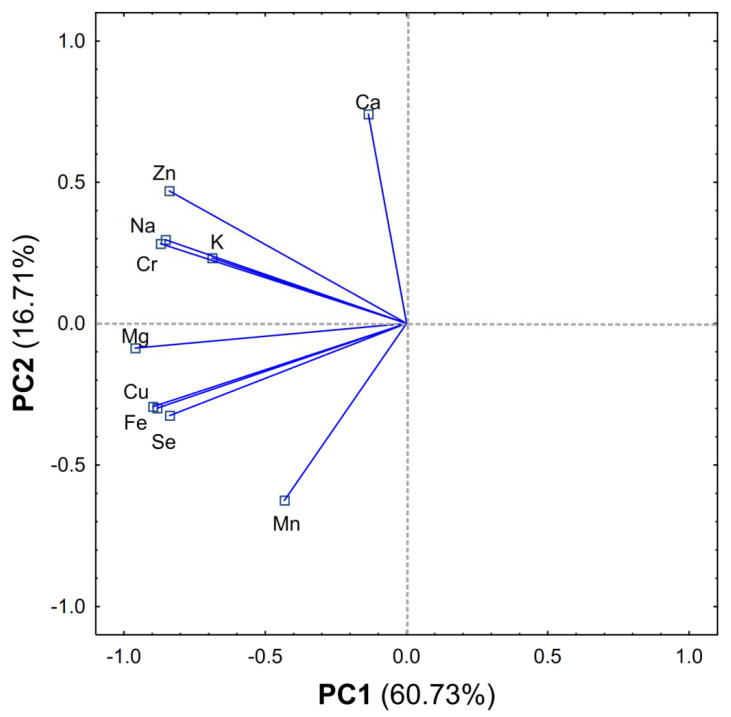
Loadings of first two components of principal component analysis (PCA), explaining together 77.44% of information in the dataset obtained.

**Figure 2:**
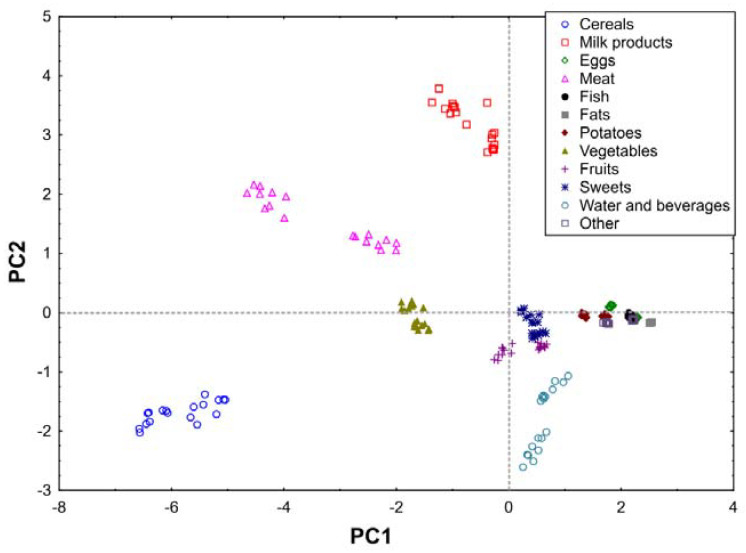
Scores of first two principal components of PCA, explaining together 77.44% of information in the obtained dataset.

From the results obtained, a matrix made of columns (elements) and rows (products) was created and subjected to PCA analysis. The PCA carried out explains 77.44% of the variability in the first two principal components (60.73% and 16.71%, respectively) (Figure 1 and Figure 2).

The first component (PC1) is related to the total intake of elements and shows the differences between cereals, meat, vegetables (mostly containing the largest element content) and the remaining products, among which attention should be paid to fats, eggs, fish, potatoes, other, which contribution to the total daily intake of determined elements was the smallest (Figure 1 and Figure 2).

The second component (PC2) reveals another group (cereals, water and beverages) which strongly correlated to the high contribution of Mn. In addition, the PC2 component separates the milk products having large amounts of Ca compared to other products (Figure 1 and Figure 2).

Moreover, cereals provide the highest amounts of Mg, Cu, Fe, Se, and contribution of meat and its products was the highest regarding the daily intake of Zn, Na, K and Cr. Vegetables should also be mentioned, as a group whose contribution to the daily intake of each element was significant (Figure 1 and Figure 2).

Comparing the results of the PCA analysis between women and men, significant differences may be observed. A different distribution of Mn intake between the genders was noticed. The daily intake of Mn among men was significantly correlated with the intake of water and beverages. On the other hand, the daily intake of this trace element among women was mainly correlated with the consumption of cereals. Additionally, a noticeably greater separation of meat in men on the graph can be observed (Figures S1 and S2), which indicates a significant contribution of this group of food products to the total intake of this metal among men. Daily intake of calcium was strongly correlated with the consumption of milk products, among both women and men. PCA revealed several visible trends in the dataset. Among men the intake of Zn, Cr Na and K was significant correlated with the consumption of meat and vegetables, whereas Mg, Se, Fe and Cu with cereals and water and beverages. Among women the intake of Mg was significantly correlated with the consumption of meat and vegetables.

## 4. Discussion

### 4.1. Dietary Intake of Macroelements with Major Food Groups

The total daily sodium intake was higher than the reference level, as expected, because many scientific data suggest a high sodium intake globally [3,12,21,22,23]. However, the results of this study indicated a much lower intake compared to other data, which revealed a daily sodium intake of above 4 g/day [12,24]. Excessive intake of sodium is a well-known nutritional problem in the adult population; however, alarmingly, a very high intake of this element has been observed recently increasingly among children [25,26], which may indicate that improper dietary habits are introduced and then consolidated starting from a very young age. Very high sodium intake is most often equated to increased consumption of processed and ultra-processed foods, except high energy density foods, and significantly associated with an increased risk for developing non-communicable diseases (NCDs) characterized by a high sodium concentration [12,26,27,28,29]. In the present study, meat and cereals were the major contributors to the daily intake of sodium. These groups of food are highly processed, and salt is added during manufacturing processes. Thus, the results obtained are in agreement with two studies performed in the United States, indicating that sodium added during food manufacturing and processing outside the home accounts for over 70% of the total daily intake [30,31]. However, in contrast to Western countries, a high amount of sodium is added at home in some Asian countries. Anderson and coworkers [32] revealed that in Guangxi, China, over 80% of sodium is added during cooking at home. Vegetables, including potatoes, formed the third food group significantly contributing to the total daily sodium intake. This is related not to the inherent low sodium content in plant products but to the salt added at home during food preparation. These findings are in agreement with the studies of Harnack and coworkers [30], indicating that salt added at home during meal preparation is a significant contributor to the overall sodium intake. The present study did not differentiate sodium addition as addition “outside the home” and “at home;” however, it can be assumed that meat and meat products and cereals are processed foods, and sodium is added to them during production. Similarly, a relatively high concentration of sodium in vegetables and potatoes is linked to the addition of salt during meal preparation at home. Daily sodium intake from other major food groups, e.g., fruits, eggs, water, and beverages or sweets and sugars, was found to be very low and insignificant in the total daily sodium intake.

The results obtained are in agreement with other data reporting insufficient potassium intake [3,21,22,24,33,34]. In addition, Sousa and Da Costa [12] found low potassium intake among women (2.3 g/day) when they assessed the adult urban population of Brazil aged 20–30. However, among Brazilian men, the daily intake was much higher (3.4 g/day) in comparison with the results of the present study. According to Welch and coworkers, potassium intake was the highest in Spain—4870 and 3723 mg/day for men and women, respectively [34]. In the United States, it was reported that only 3% of adults and 10% of children under the age of 5 meet the AI value of this element [35,36]. Moreover, the 2015–2020 Dietary Guidelines for Americans identify potassium as a “nutrient of public health concern” [37]. The results of this study also indicated that the mean potassium intake is highly inadequate among both women and men and only 68% and 73% of the AI value are met, respectively.

The contribution of foods to the total potassium intake differs significantly between various geographical origins. In general, meat and meat products and cereals are the main contributors to potassium intake in Europe. However, substantial differences are seen between countries and regions. In Greece, the main contributors to potassium intake are vegetables and fruits (39% of the total intake), whereas they contribute only 17.5% of the recommended level of potassium in Nordic countries [34]. In the United Kingdom, vegetables and potatoes are the largest contributors to potassium intake, providing 24.5% of the overall daily intake [38]. According to recent Polish data, potatoes are considered the main contributor to potassium intake [18]. The current results revealed that potatoes were not the main contributors to potassium intake in either women or men, which is in contrast to the aforementioned Polish data [18]. Since meat was the major contributor to the daily intake of potassium among men, it may indicate a changing trend in the dietary habits of the population with increasing consumption of meat and its products, especially among men. On the other hand, vegetables were found to provide the highest amounts of potassium among women. It should be also noted that, among both genders, fruits and cereals contributed significantly to the daily intake of this macroelement, which proved the important role of these groups of foods regarding dietary intake of potassium among young adults in Poland. A significant contribution of foods of plant origin to the daily intake of potassium was also shown in studies performed in Greece [34] and UK [38].

Although the daily intake of both minerals is still incorrect, the Na:K ratios calculated for men and women were 0.82 and 1.12, respectively, which were much lower in comparison to the US data (1.32 among women and 1.45 among men) [33], as well as the data obtained for the Polish population in a previous study [3]. This may indicate the positive changes in the dietary habits especially with respect to sodium intake, as this parameter is close to 1.0 as recommended by the NHANES III (1988–1994) (National Health and Nutrition Examination Survey) study, in which Yang and coworkers [39] found a beneficial protective effect for the usual Na:K ratio <1.0 and a reduced risk of cardiovascular diseases (CVD) and all-cause mortality.

Such a low magnesium intake, as indicated in the present research, can be found in the scientific literature, e.g., in studies from Italy (262 mg/day) [21] or the United States (234 and 268 mg/day, for women and men, respectively) [40]. Apparently, the dietary intake of magnesium in the previous years was much higher, exceeding 300 mg/day [22,41,42]. In turn, Sousa and Da Costa [12] showed in their study that the mean magnesium intake among Brazilian adults who were aged 20–30 was 441.6 mg/day among women and 607.3 mg/day among men. Such a high dietary intake of magnesium is very rarely observed in scientific data from other parts of the world. In a recent large study on mineral intake among adults conducted in Eastern Poland using a market basket method, the mean magnesium intake was shown to be within the range of 274–492 mg/day [3]. These indicate that the levels revealed by the results of the present study are much lower. Very low magnesium intake may be very harmful for the general population and may cause several health problems, including CVD and renal failure and increase the prevalence of obesity and metabolic syndrome [43,44,45]. According to Schwalfenberg and Genuis [45], there are many reasons for inadequate magnesium intake, which include: low levels of Mg in many processed foods and some non-organic foods; low magnesium content in common staples such as meat, sugar, and white flour; significant loss of magnesium during cooking and boiling of food products; and use of demineralized water for drinking.

Green leafy vegetables, such as spinach, legumes, nuts, seeds, and whole grains, are considered as good sources of magnesium. Breakfast cereals and other fortified foods with artificially added magnesium salts may also significantly contribute to the overall daily magnesium intake. On the other hand, some methods of food processing, such as refining of grains in ways that remove the nutrient-rich germ and bran, substantially lower the content of this element [46,47]. In addition, tap, mineral, and bottled water may provide significant amounts of magnesium, but the amount of mineral in water varies by source and brand (ranging from 1 to >120 mg/L) [48]. According to previously published Polish data [18], cereals and selected vegetables are major contributors to total magnesium intake. This is partially in agreement with the results of the current investigation. Cereals were found to be the major contributor to the daily intake of magnesium, followed by meat, vegetables, fruits and milk and diary. Although the role of cereals and vegetables as important contributors to the intake of magnesium is well known, this study sheds new light on the role of meat and its products in the overall dietary intake of magnesium. This group of food is considered highly processed and rather poor in magnesium [45]. However, their high consumption contributes to significantly high magnesium intake, which was proved in the present study On the other hand, although tap water from Lublin is rich in calcium and magnesium, the contribution of water and beverages (prepared using tap water) to the overall magnesium intake was relatively low: 4.4% for women and 6.6% for men.

Although it is widely known that calcium intake is insufficient in many countries, very low consumption is, rather, rarely observed in the scientific literature. Raghunath and coworkers [22], who investigated the dietary intake of several metals among the adult Indian population, revealed that calcium intake was only 367 mg/day. However, the Indian diet was mostly based on plant foods, and therefore, such low calcium intake was expected. Despite being highly insufficient and below the recommended values, the dietary intake of calcium reported during the last 20 years was generally much higher compared to the results of the present study. Lombardi-Boccia and coworkers [21] determined that calcium intake with Italian daily food rations was 738 mg/day, while in Spain the mean intake was 1267 mg/day [41], in Poland 712–1365 mg/day (depending on the investigated group of adults) [3], in South Australia 1097 mg/day [49], in the United Kingdom 1007 and 777 mg/day among men and women, respectively [50], and in Brazil 1143 mg/day among females over the age of 50 [12].

In general, calcium intake is associated with the consumption of milk and dairy products, which are a rich source of this element. Other dietary sources of calcium are cereals, especially those fortified with calcium salts, and vegetables such as kale, broccoli, and watercress, which provide between 100 and 150 mg/100 g [51]. However, individual food patterns and dietary habits were shown to determine the major calcium sources among specific populations. In developed countries, energy intake from dairy products represents around 14% of the total energy intake, whereas in the developing countries it represents only 4% [52]. Therefore, in Asian or African countries, the main calcium suppliers are non-animal foods such as vegetables, legumes, and grains rather than dairy products, which are the primary calcium source in Europe and the United States. Since plant foods have low calcium content, the daily intake of this element in Asia or Africa is mostly lower compared to the developed countries [51,52]. Moreover, calcium bioavailability from plant foods is significantly lower compared to dairy products, which also diminishes the body’s calcium balance. In the United States, Netherlands, and the United Kingdom, 72, 58, and 43% of daily calcium level are derived from dairy products, whereas in China, only around 7% of total calcium comes from dairy products, while the majority intake is from vegetables (30.2%) and legumes (16.7%) [50,51,53,54]. The results of this study are in agreement with these data, as milk and dairy products were by far the major contributors to the daily calcium intake. This was mostly due high concentration of calcium in milk and diary in combination with high consumption of this group of products. However, still, among both genders, the daily calcium intake was far below the recommended level of intake. Hard water is also considered a potential source of dietary calcium [51]. However, studies on an inverse association between water hardness and cardiovascular diseases provide inconclusive results [51,55,56]. Although tap water from the Lublin region is considered hard (an average hardness over 20°dH), the contribution of water and beverages to the total calcium supply was relatively low—only 2.3% and 3.1% among women and men, respectively—which may be partly related to the boiling of water, causing some of the calcium compounds to precipitate. However, it should be remembered that in the present study the intake of calcium with plain water was not evaluated and, therefore, the total intake of this element may be underestimated.

### 4.2. Dietary Intake of Trace Elements with Major Food Groups

For Cu, Zn, Fe, and Se, the adequate EAR values were used as a reference, while for Mn the AI reference values for the Polish adult population were used [18]. As Polish standards do not form the reference intake for chromium, appropriate US reference intake standards were used [57].

Daily intake of copper among both genders was far above the reference value for the Polish population. According to scientific data, the dietary intake of this trace metal in a majority of the countries is not associated with any health risk as the daily intake exceeds 1 mg [12,58,59,60,61]. In a previous study in the Lublin region, the mean copper intake was 2.07–4.30 mg/person/day [3], so the levels shown by the current investigation are much lower, yet still higher than the recommended values.

Offal (liver), wheat germ and bran, oatmeal, nuts, some seafood (oyster), cocoa, and sunflower seeds are the food products rich in copper. Sometimes, tap water may be considered a rich source of copper, especially when fittings are made of copper alloys [62,63]. However, low consumption of the aforementioned products leads to an insignificant overall copper intake. According to EFSA (European Food Safety Authority), cereals are the most important contributor to copper intake, not only because of significantly high copper concentration but also because of the high consumption of these products (e.g., bread) and a large variety of other products. The second most important group of products was meat and meat products, which provided 19% of daily copper intake among men and 16% among women. The contribution of water and water-based beverages to copper intake was on average 12% [62]. According to recent Polish data, among adults, cereals were considered the most important contributor to copper intake, which provided 30% of the daily copper intake, followed by potatoes (15%), vegetables (13%), and meat and meat products (11%) [18]. The results of the present study are in full agreement with the European and Polish data, as cereals provided the highest amounts of copper in the daily diet, followed by vegetables, meat and fruits. In addition, it was revealed that the contribution of products based on tap water (water and beverages) to the daily copper intake was very low—2.1% and 2.9% among women and men, respectively, which confirms the estimation of de Romana and coworkers [63] that foods account for over 90% of copper intake in adults when the concentration of this metal in water is low (<0.1 mg/L).

Zinc is an essential trace element, the dietary intake of which is usually above the recommended values in a majority of the countries. Studies conducted in recent years revealed that zinc intake was 9.8 mg/day in China [60], 10.1–15.2 mg/day in Spain [58], 12 mg/day in Italy [7], 10.5–18.2 mg/day in Poland [3], and 10 and 14.6 mg/day in Brazil among adult women and men, respectively [12]. According to EFSA, the mean zinc intake in European countries ranges between 8 and 14 mg/day in adults [64]. The results of the present study are in agreement with these data. This suggests that, based on the mean intake, the risk of zinc deficiency is relatively low among Polish adults.

In Europe, the main food groups contributing to zinc intake were meat and meat products, grains and grain-based products, and milk and dairy products. Vegetables (in Italy) and fish and fish products (in Italy and Sweden) were also considered as significant sources of zinc [64]. Animal-derived foods are the richest source of absorbable zinc, especially organs and flesh of mammals, fowl, fish, and crustaceans. Cereals, nuts, and legumes have lower and less efficiently absorbed zinc compared to animal-source food. Of the plant-based foods, fruits and vegetables, starchy roots, and tubers are low in zinc [65,66,67]. Moreover, since the concentration of zinc in plant-based foods is affected by that in soil, local food-composition tables or direct chemical determinations should be used to evaluate the intake of this element [67]. Results of the present determinations revealed that meat and cereals were the major contributors to the daily intake of zinc. Interestingly, significant amounts of zinc in the daily diet of both, women and men, came from milk and dairy products. On the other hand, the contribution of vegetables to the intake of this trace element was low.

Similar to zinc, manganese is another trace element, the dietary intake of which, according to scientific data, does not pose any health risk and is usually higher than the recommended intake level. Dietary intake reported in various countries was as follows: 5.9 mg/person/day in China [60], 2.07–2.81 mg/person/day in the United States [68], 3.7 mg/person/day in Sweden [42], 3.5–7.9 mg/person/day in Poland [3], and 9.3 and 25.7 mg/person/day among adult women and men, respectively, in Brazil [12]. According to EFSA, the dietary intake of manganese in a majority of European countries was within 2–6 mg/day, with an average value of 3 mg/day [69]. The results of the current investigation also support these data.,

Nuts, chocolate, cereal-based products, crustaceans and mollusks, pulses, and fruits and fruit products are considered rich sources of manganese [70,71]. Tea (black, green, Yerba-Mate) is also a rich source of manganese, as one cup of this beverage (200 mL) contains 1.3 mg of Mn [12,69]. Cereals, tea, coffee, vegetables, and fruits are considered major manganese sources in the general population [18,69]. In some countries, tea infusions (black or green) have been found to contribute to over 30% of the daily manganese consumption [72,73,74]. According to da Sousa and coworkers, a very high intake of Mn among Brazilian adults (far above the data reported for other countries) may be related to the very high consumption of Yerba-Mate tea which is rich in manganese [12,75]. In the present study cereals (among women) and water and beverages (among men) were proved to be the major contributors to the daily intake of manganese, which confirms their significant role (especially tea), as important sources of this trace element in the daily diet.

Iron is a trace element, the dietary intake of which is a special health concern, especially among women of childbearing age [3,12,76]. A recent study performed in Brazil revealed that the prevalence of iron deficiency was only 11% in women aged 20–50 years and intake in women in the fifth percentile was within the reference value. Scientific data indicated that the daily iron intake was 22.7 mg in China [60], 13 mg in Lebanon [9], 16 mg in Sweden [42], 11.6 mg in Brazil (women aged 20–50) [12], and 13.5 mg among women and 18.1 mg among men in Poland [3]. The chemical determinations in the present study also revealed that the total iron intake is well above the recommended EAR values, for both women and men. Although the above data suggest that iron consumption in many countries is more than the recommended intake levels, it is estimated that worldwide the number of people at risk of developing iron deficiency or anemia is as high as 30%, while in the United States it is 2–5%, which is still a very high number [77,78]. Despite the fact that the groups with the highest risk of developing anemia are pregnant women, infants, and children aged 6 months to 4 years, low consumption of products rich in highly absorbable iron may lead to significant depletion of endogenous iron [79].

The main sources of iron in the diet, due to the much higher bioavailability of the heme form, are animal products—pork and beef, liver, kidneys, poultry, and fish. According to Grajeta [80], products containing a non-heme form of iron, such as spinach, wheat germ, lettuce, legume seeds, or bread, can also be a valuable source of iron, only if the food ration is properly composed and balanced, providing nutrients that increase the absorption of this form of the micronutrient. In Poland, based on the amount of consumption, the main source of iron in the diet is identified as cereal products, which supplies 32–39% of the total amount of iron in the food ration, followed by meat and fish providing 22–27%, potatoes 15–20%, and vegetables and fruits 9–14% [81]. In a study from the United States, Guthrie and Picciano [82] reported that cereal products contributed to 43% of the total amount of iron in the diet, followed by meat and fish products 22%, vegetables and seeds 20%, eggs 3%, fruits 3%, and other products 9%. The results of the present study are partially in agreement with these data, as cereals and meat were the main contributors to the daily iron intake. However, the contribution of meat and its products to iron intake was much lower compared to the US data and previous data from Poland. In contrast to data obtained in other countries fish, because of a very low consumption, were not found to be as significant a contributor to the daily intake of this trace element.

For over 40 years, chromium was considered as a trace metal essential to humans. However, in recent times, its vital role has been under debate [83,84]. According to Vincent, nutritional research clearly indicates that chromium should not be considered as an essential element and dietary intake standards should not be established for this element as done for other essential nutrients [85]. However, the updated Polish nutritional standards indicate the reference consumption of chromium at the AI level and, therefore, this metal has been considered as an essential nutrient. Scientific data from the last 20 years show that the chromium intake level varies in the range of 40–154 µg/person/day in the United States and European countries and is far above the recommended intake level [86,87,88], which was confirmed in the present study. The results of the present determinations agree with other European data, suggesting that cereals are the main contributor to the overall chromium intake, followed by meat, milk and dairy products, and vegetables [86,88,89]. However, these data, for the first time, clearly indicated that sweets and sugars are a food group significantly contributing to the overall chromium consumption.

Foods originating from plants and animals are the main source of selenium for humans. The amount of selenium in the diet varies depending on the agricultural conditions and place of animal breeding, as well as based on the concentration of selenium in the soil [90,91]. The daily dietary intake of selenium also depends mainly on the region. In Suzhou, China, characterized by moderate selenium deficiency, the daily intake was established at 43.9 µg/person [92], a level slightly below the AI value. On the other hand, in Europe, the selenium intake level has been observed to be much higher in recent years, ranging between 39 µg/person/day in the United Kingdom [89] and 104 µg/person/day in Italy [21]. The results of this study revealed that the mean selenium intake is close to the content present in Italian diets and far above the recommended level of intake among both women and men. Thus, the levels determined in the study are much higher compared to the previous studies performed in Poland, which established the selenium intake level at 54–66 µg/person/day (duplicate diet study) [88] and 37–76.4 µg/person/day (market basket study) [3]. Daily selenium intake of 400–800 µg/person/day is considered safe. Therefore, the dietary intake of this trace element revealed in the present research is appropriate, as it is higher than the EAR value, and much below levels considered being toxic for humans (1.5–1.6 mg/day) [93].

In the United Kingdom, meat and poultry were considered as the most important foods, accounting for 34% of total selenium intake, followed by cereals (23%), milk products (15%), fish (13%), eggs (8%), and vegetables (8%) [89]. In the Netherlands, the main contributors to the overall daily selenium intake were cereals (24%), poultry and eggs (15%), and milk products and bread (13%) [94]. According to the studies performed by Marzec in Poland 20 years ago [88], the best sources of this element were cereals (38%), meat and meat products (24%), vegetables (12%), milk and dairy (6%), and eggs, fish, and fruits (5% each). Obtained results revealed that cereals, meat and vegetables were the major contributors to the daily selenium intake, which is in agreement with data from other countries. The present results also indicated water and beverages to be an important group of food products, the contribution of which to the daily intake of selenium is significant.

## 5. Conclusions

The present research combined a 24-dietary recall technique with analytical determinations of elements using F-AAS and ICP-OES methods, which together enabled the evaluation of intake and pointed to the main food groups whose contribution to the total intake of particular elements was significant. The results obtained revealed a very high intake of sodium and a very low intake of calcium. Dietary intake of trace elements was within the range of reference values in the subjects, with cereals being the major source of a majority of those elements, while meat, vegetables, and milk products for Ca were the main contributors to the daily dietary intake of macroelements. PCA supported analytical determinations and revealed significant correlations between the daily intake of Zn, Cr, Na and K and the consumption of meat and vegetables as well as Mg, Se, Fe and Cu and the consumption of cereals.

## Figures and Tables

**Table 1:** Characterization of the studied population.

Parameter	Women (*N* = 274)	Men (*N* = 305)
Age (years)	24 ± 4	24 ± 4
Physical activity	Moderate	Moderate
Height (m)	1.58 ± 0.17	1.74 ± 0.19
Weight (kg)	58 ± 6.4	74 ± 8.5
BMI (kg/m^2^)	23.2 ± 1.90	24.4 ± 2.75

BMI—Body Mass Index.

**Table 2:** Selective parameters of the applied spectroscopic determinations.

Parameter	Element									
	Na	K	Mg	Ca	Cu	Zn	Mn	Fe	Cr	Se
Reference value (mg/kg)	6300	10,260	752.3	3522	2.94	24.0	9.02	22.9	0.15	0.20
Determined value (mg/kg)	6178	10,722	754.2	3452	2.97	23.1	8.80	20.7	0.17	0.21
SD	249.4	884	39.3	202.6	0.15	1.97	0.36	1.76	0.017	0.017
RSD (%)	4.04	8.24	5.21	5.87	5.05	8.53	4.09	8.50	10.0	8.10
Recovery (%)	98.1	104.5	100.3	98.0	101.0	96.3	97.6	90.4	113.3	105
LOD (µg/kg)	82.0	65.0	37.0	182	182	40.0	171.0	161.0	1.2	2.2
LOQ (µg/kg)	295	279.5	135.7	600.6	637.5	148.4	607.3	536.3	4.3	7.5

SD—Standard Deviation; RSD—Relative Standard Deviation; LOD—Limit of Detection; LOQ—Limit of Quantification.

**Table 3:** Average daily intake of foods by food groups among women and men.

Food Group	Women			Men		
	Amount (g)	Energy (kcal)	Composition (%)	Amount (g)	Energy (kcal)	Composition (%)
Cereals	224.1 ± 20.8	653.6 ± 79.2	baking 51.5 cereal flakes 14.7 grits 12.5 rice 11.5 pasta 9.80	284.8 ± 25.3	770.1 ± 84.2	baking 66.6 rice 12.9 pasta 9.40 cereal flakes 6.35 grits 4.75
Milk and diary	276.9 ± 25.7	303.9 ± 35.4	milk 45.1 cheese 30.4 yogurts 23.2 sour cream 1.3	232.3 ± 22.4	327.1 ± 29.8	cheese 47.0 milk 31.2 yogurts 18.9 sour cream 2.90
Eggs	18.2 ± 2.11	21.4 ± 1.92	scrambled eggs 48.1 boiled eggs 38.7 omelette 13.2	45.5 ± 5.17	53.1 ± 7.12	scrambled eggs 60.9 boiled eggs 33.2 omelette 5.90
Meat and meat products	191.7 ± 20.8	406.5 ± 47.2	pork 39.0 poultry 37.6 beef 23.4	283.5 ± 31.4	621.5 ± 56.4	pork 42.0 beef 30.4 poultry 27.6
Fish	17.5 ± 2.14	21.4 ± 2.72	tuna 44.2 cod 38.6 salmon 12.0 herring 4.0 pike 1.2	20.0 ± 3.18	36.7 ± 4.12	mackerel 47.0 salmon 35.3 tuna 8.90 sardine 8.80
Fats and oils	14.0 ± 1.34	116.7 ± 11.4	butter 35.2 rapeseed oil 32.5 sunflower oil 15.7 olive oil 11.1 margarine 3.29 linseed oil 2.21	25.1 ± 2.91	209.7 ± 18.4	rapeseed oil 38.2 butter 32.6 sunflower oil 20,2 lard 6.90 olive oil 2.10
Potatoes	50.1 ± 6.55	38.6 ± 4.22		82.5 ± 9.52	63.5 ± 7.48	
Vegetables	384.1 ± 45.2	90.9 ± 12.5	tomatoes 30.1 pepper 19.4 carrot 10.9 cabbage 9.95 beetroot 7.85 cauliflower 6.82 broccoli 6.64 lettuce 2.17 celery 1.16 others 5	343.8 ± 38.4	72.6 ± 9.15	tomatoes 32.2 carrot 22.3 pepper 15.6 cucumber 11.2 onion 4.05 cabbage 3.58 broccoli 2.50 pumpkin 1.30 leek 1.27 lettuce 1.05 others 5
Fruits	227.2 ± 35.6	140.5 ± 18.8	apples 34.2 bananas 33.6 oranges 7.65 mandarins 5.92 pears 4.14 peaches 3.42 raspberries 3.05 watermelons 1.86 kiwi 1.48 raisins 1.32 nectarines 1.25 others 1.44	192.2 ± 26.2	114.4 ± 17.8	apples 40.5 bananas 28.9 oranges 10.1 mandarins 5.92 pears 5.56 grapefruit 3.10 grapes 1.55 red currants 1.85 dried dates 1.36 others 1.16
Sweets and sugars	62.4 ± 10.2	236.9 ± 31.8	cakes and cookies 53.4 bars 14.3 sugar 13.4 chocolate 12.5 honey 1.70 others 4.70	69.1 ± 12.4	251 ± 40.8	cakes and cookies 60.5 sugar 23.4 bars 10.6 chocolate 4.80 honey 0.70
Water and beverages	638.7 ± 75.4	33.8 ± 4.52	black tea (infusion) 56.8 soups (only water) 18.8 fruit juices 11.1 coffee (infusion) 11.0 sweet beverages 2.30	713.1 ± 100.2	52.3 ± 6.18	black tea (infusion) 53.4 coffee (infusion) 17.0 soups (only water) 13.2 fruit juices 11.8 sweet beverages 3.27 bear 1.33
Other products	34.5 ± 5.52	139 ± 15.8	jams (no added sugar) 32.4 sauces 22.8 hooves 16.9 seeds (pumpkin/sunflower) 10.6 nuts 5.50 snacks 4.24 pudding 2.56 almonds 2.11 tomato concentrate 1.94 cocoa 0.95	18.4 ± 3.11	98.7 ± 10.2	sauces 56.3 jams (no added sugar) 22.0 pancakes 13.4 almonds 2.78 nuts 2.25 pudding 1.68 cocoa 1.10 others 0.49
Total	2139 ± 264	2203 ± 331		2310 ± 351	2671 ± 355	

**Table 4:** Daily intake of macroelements with particular food groups. Each value represents mean ± SD. Means not sharing the same letter in a column are significantly different at *p* ≤ 0.05. Means underlined are significantly different between women and men at *p* ≤ 0.05.

Food Group	Na (mg)		K (mg)		Mg (mg)		Ca (mg)	
	Women	Men	Women	Men	Women	Men	Women	Men
Cereals	**588 ± 31.3 ^g^** 550–642	**634 ± 32.6 ^f^** 590–674	**290 ± 17.3 ^e^** 261–311	**253 ± 12.4 ^d^** 236–270	**63.9 ± 4.93 ^g^** 58.3–71.7	**66.8 ± 4.78 ^g^** 58.4–73.2	**11.4 ± 1.67 ^a^** 9.22–14.2	**13.8 ± 0.52 ^cd^** 13.0–14.7
Milk and diary	**219 ± 10.5 ^e^** 205–234	**324 ± 20.2 ^d^** 293–356	**289 ± 19.1 ^e^** 262–316	**201 ± 20.3 ^c^** 170–230	**22.5 ± 1.32 ^e^** 20.1–24.5	**20.9 ± 2.97 ^d^** 17.3–26.5	**284 ± 34.0 ^c^** 247–359	**322 ± 16.1 ^f^** 301–354
Eggs	**18.0 ± 1.56 ^abc^** 15.1–20.3	**37.6 ± 1.73 ^ab^** 34.9–39.5	**17.8 ± 2.01 ^a^** 15.6–21.5	**46.0 ± 4.99 ^b^** 37.4–52.3	**1.23 ± 0.18 ^b^** 1.00–1.55	**3.26 ± 0.60 ^a^** 2.32–3.96	**3.02 ± 0.46 ^a^** 2.55–3.92	**21.0 ± 2.87 ^d^** 18.1–26.6
Meat and meat products	**671 ± 66.7 ^h^** 582–760	**1012 ± 69.6 ^g^** 912–1125	**460 ± 60.0 ^f^** 356–523	**672 ± 39.8 ^h^** 612–719	**37.9 ± 6.03 ^d^** 29.5–46.2	**36.5 ± 3.73 ^f^** 31.1–42.2	**9.62 ± 0.49 ^a^** 9.14–10.2	**7.50 ± 0.49 ^abc^** 6.76–8.06
Fish	**39.6 ± 1.55 ^bc^** 37.2–42.5	**53.1 ± 3.93 ^b^** 46.2–57.4	**26.0 ± 1.69 ^ab^** 24.1–28.9	**32.5 ± 3.25 ^ab^** 28.1–36.8	**2.37 ± 0.21 ^bc^** 1.98–2.68	**3.21 ± 0.30 ^a^** 2.82–3.72	**0.67 ± 0.09 ^a^** 0.55–0.82	**0.49 ± 0.04 ^a^** 0.42–0.53
Fats and oils	**0.08 ± 0.01 ^a^** 0.06–0.10	**0.05 ± 0.01 ^a^** 0.04–0.07	**0.32 ± 0.03 ^a^** 0.28–0.35	**1.50 ± 0.21 ^a^** 1.14–1.78	**0.03 ± 0.01 ^b^** 0.02–0.05	**0.08 ± 0.01 ^a^** 0.06–0.09	**0.15 ± 0.02 ^a^** 0.11–0.17	**0.90 ± 0.04 ^a^** 0.84–1.00
Potatoes	**104 ± 10.1 ^d^** 88.3–118	**162 ± 9.48 ^c^** 152–178	**123 ± 12.0 ^d^** 105–141	**195 ± 19.0 ^c^** 160–219	**6.30 ± 0.35 ^ac^** 5.72–6.79	**10.0 ± 0.37 ^e^** 9.55–10.7	**1.02 ± 0.11 ^a^** 0.82–1.18	**1.48 ± 0.1 ^ab^** 1.34–1.63
Vegetables	**360 ± 22.4 ^f^** 320–390	**372 ± 25.0 ^e^** 333–415	**650 ± 36.4 ^g^** 608–705	**456 ± 44.1 ^g^** 410–528	**34.2 ± 3.45 ^d^** 28.5–40.1	**21.5 ± 2.10 ^d^** 18.8–24.1	**43.1 ± 2.56 ^b^** 38.7–47.1	**31.0 ± 1.40 ^e^** 28.1–32.9
Fruits	**3.70 ± 0.26 ^ab^** 3.29–4.12	**1.08 ± 0.19 ^a^** 0.84–1.34	**465 ± 35.8 ^f^** 406–512	**273 ± 17.8 ^d^** 250–303	**30.0 ± 2.39 ^f^** 26.2–33.5	**17.2 ± 1.22 ^c^** 15.6–18.9	**5.79 ± 0.57 ^a^** 5.12–6.84	**6.36 ± 0.64 ^abc^** 5.34–6.98
Sweets and sugars	**48.1 ± 4.91 ^c^** 40.7–56.1	**55.9 ± 7.91 ^b^** 42.5–67.6	**68.7 ± 5.28 ^c^** 58.2–75.6	**84.8 ± 10.8 ^e^** 70.3–105	**8.18 ± 0.19 ^a^** 7.87–8.55	**14.7 ± 0.73 ^bc^** 13.6–15.7	**9.87 ± 1.38 ^a^** 8.31–12.6	**8.43 ± 0.40 ^bc^** 7.65–8.86
Water and beverages	**4.16 ± 0.14 ^ab^** 3.94–4.29	**4.52 ± 0.35 ^a^** 3.91–5.03	**113 ± 10.3 ^d^** 101–132	**159 ± 21.3 ^f^** 132–198	**9.88 ± 1.34 ^a^** 8.11–12.3	**13.8 ± 1.00 ^b^** 12.6–15.9	**8.90 ± 0.30 ^a^** 8.39–9.25	**13.1 ± 1.32 ^c^** 11.7–15.1
Other products	**47.6 ± 4.81 ^c^** 41.2–55.7	**21.6 ± 2.24 ^ab^** 17.9–24.6	**60.5 ± 3.15 ^bc^** 56.0–65.6	**21.6 ± 2.08 ^ab^** 18.1–24.6	**7.89 ± 0.85 ^a^** 6.92–9.34	**2.49 ± 0.27 ^a^** 2.05–2.82	**7.89 ± 0.37 ^a^** 7.46–8.56	**2.49 ± 0.19 ^ab^** 2.28–2.80
Total	**2103 ± 94.0**	**2679 ± 87.0**	**2564 ± 99.0**	**2396 ± 89.0**	**224 ± 3.00**	**210 ± 7.00**	**385 ± 36.0**	**429 ± 18.0**
EAR/AI [22]	1500	1500	3500	3500	255	330	800	800

EAR—Estimated Average Requirement; AI—Adequate Intake.

**Table 5:** Daily intake of trace elements with particular food groups. Each value represents mean ±SD. Means not sharing the same letter in a column are significantly different at *p* ≤ 0.05. Means underlined are significantly different between women and men at *p* ≤ 0.05.

Food Group	Cu (mg)		Zn (mg)		Mn (mg)		Fe (mg)		Cr (µg)		Se (µg)	
	Women	Men	Women	Men	Women	Men	Women	Men	Women	Men	Women	Men
Cereals	**0.331 ± 0.035 ^h^** 0.291–0.376	**0.377 ± 0.028 ^h^** 0.331–0.412	**2.15 ± 0.21 ^g^** 1.85–2.46	**2.32 ± 0.14 ^d^** 2.09–2.45	**1.68 ± 0.11 ^g^** 1.52–1.87	**1.57 ± 0.09 ^e^** 1.41–1.68	**4.70 ± 0.33 ^g^** 4.22–5.15	**6.95 ± 0.44 ^i^** 6.16–7.51	**34.3 ± 3.49 ^d^** 28.2–38.9	**41.4 ± 3.89 ^d^** 35.6–47.2	**29.9 ± 5.45 ^h^** 25.2–42.4	**31.0 ± 5.32 ^h^** 22.8–38.5
Milk and diary	**0.034 ± 0.004 ^cd^** 0.028–0.038	**0.030 ± 0.005 ^a^** 0.024–0.039	**1.92 ± 0.13 ^f^** 1.71–2.14	**2.24 ± 0.08 ^d^** 2.14–2.35	**0.04 ± 0.003 ^ab^** 0.03–0.04	**0.032 ± 0.004 ^ab^** 0.027–0.038	**0.30 ± 0.05 ^ab^** 0.24–0.36	**0.568 ± 0.060 ^cde^** 0.450–0.650	**19.1 ± 1.61 ^f^** 17.0–21.8	**36.0 ± 4.44 ^e^** 28.9–42.5	**4.99 ± 0.55 ^bc^** 4.11–5.72	**9.18 ± 0.77 ^be^** 8.28–10.4
Eggs	**0.010 ± 0.002 ^ab^** 0.008–0.013	**0.022 ± 0.002 ^acd^** 0.018–0.025	**0.20 ± 0.03 ^abc^** 0.16–0.25	**0.41 ± 0.03 ^c^** 0.360–0.450	**0.004 ± 0.001 ^a^** 0.003–0.006	**0.018 ± 0.004 ^a^** 0.014–0.025	**0.26 ± 0.03 ^a^** 0.21–0.29	**0.530 ± 0.056 ^cd^** 0.440–0.590	**1.47 ± 0.25 ^bc^** 1.06–1.75	**5.64 ± 0.43 ^a^** 5.12–6.26	**0.60 ± 0.08 ^a^** 0.47–0.70	**2.13 ± 0.27 ^ac^** 1.62–2.36
Meat and meat products	**0.132 ± 0.016 ^e^** 0.116–0.158	**0.138 ± 0.010 ^e^** 0.122–0.151	**2.46 ± 0.22 ^h^** 2.11–2.74	**3.39 ± 0.39 ^f^** 2.80–3.82	**0.14 ± 0.007 ^c^** 0.13–0.15	**0.101 ± 0.014 ^ab^** 0.075–0.118	**1.80 ± 0.26 ^f^** 1.45–2.26	**2.20 ± 0.153 ^h^** 1.88–2.34	**23.8 ± 2.09 ^g^** 19.8–26.2	**40.6 ± 4.13 ^d^** 34.7–46.2	**6.70 ± 0.93 ^cd^** 5.33–8.22	**17.3 ± 1.19 ^f^** 15.3–18.5
Fish	**0.006 ± 0.001 ^ab^** 0.004–0.007	**0.009 ± 0.001 ^bc^** 0.007–0.011	**0.8 ± 0.01 ^ad^** 0.06–0.09	**0.087 ± 0.015 ^ab^** 0.060–0.110	**0.003 ± 0.0008 ^a^** 0.002–0.004	**0.005 ± 0.0007 ^a^** 0.003–0.005	**0.14 ± 0.02 ^ac^** 0.12–0.17	**0.213 ± 0.027 ^ab^** 0.170–0.250	**6.07 ± 0.65 ^a^** 5.22–7.11	**2.65 ± 0.39 ^ab^** 2.24–3.28	**1.52 ± 0.25 ^ab^** 1.27–1.90	**0.91 ± 0.13 ^a^** 0.76–1.11
Fats and oils	**0.0003 ± 0.0001 ^a^** 0.0002–0.0005	**0.001 ± 0.0002 ^b^** 0.0008–0.0014	**0.001 ± 0.0002 ^d^** 0.0008–0.001	**0.002 ± 0.0003 ^a^** 0.002–0.003	**<LOQ**	**<LOQ**	**0.006 ± 0.001 ^c^** 0.005–0.008	**0.017 ± 0.003 ^a^** 0.014–0.023	**0.30 ± 0.06 ^c^** 0.22–0.37	**0.27 ± 0.03 ^b^** 0.22–0.32	**0.87 ± 0.11 ^a^** 0.67–0.98	**1.31 ± 0.14 ^a^** 1.11–1.51
Potatoes	**0.023 ± 0.003 ^bc^** 0.020–0.031	**0.033 ± 0.003 ^a^** 0.028–0.038	**0.16 ± 0.016 ^ab^** 0.14–0.18	**0.234 ± 0.025 ^bc^** 0.200–0.280	**0.07 ± 0.003 ^b^** 0.06–0.07	**0.116 ± 0.010 ^abc^** 0.102–0.128	**0.29 ± 0.03 ^ab^** 0.25–0.35	**0.324 ± 0.043 ^bc^** 0.260–0.380	**4.09 ± 0.27 ^ab^** 3.75–4.48	**3.13 ± 0.45 ^ab^** 2.47–3.78	**2.39 ± 0.27 ^ab^** 2.02–2.78	**4.30 ± 0.30 ^cd^** 3.82–4.67
Vegetables	**0.170 ± 0.014 ^g^** 0.150–0.191	**0.145 ± 0.007 ^e^** 0.132–0.155	**0.88 ± 0.07 ^e^** 0.78–0.99	**0.704 ± 0.044 ^e^** 0.620–0.770	**0.47 ± 0.01 ^d^** 0.46–0.49	**0.383 ± 0.024 ^d^** 0.351–0.414	**1.30 ± 0.11 ^e^** 1.14–1.45	**1.90 ± 0.18 ^g^** 1.60–2.15	**12.3 ± 1.63 ^e^** 10.2–14.8	**17.1 ± 1.85 ^c^** 14.5 - 20.2	**15.4 ± 0.92 ^f^** 14.5–16.9	**22.4 ± 1.52 ^g^** 20.1–25.3
Fruits	**0.143 ± 0.019 ^e^** 0.121–0.172	**0.114 ± 0.007 ^g^** 0.107–0.129	**0.27 ± 0.03 ^bc^** 0.24–0.32	**0.149 ± 0.020 ^ab^** 0.120–0.180	**0.55 ± 0.002 ^e^** 0.53–0.59	**0.145 ± 0.012 ^bc^** 0.131–0.165	**0.48 ± 0.04 ^bd^** 0.42–0.55	**0.790 ± 0.048 ^e^** 0.720–0.870	**5.08 ± 0.35 ^a^** 4.45–5.46	**5.06 ± 0.56 ^a^** 4.18–5.69	**10.8 ± 1.29 ^e^** 9.23–12.3	**11.6 ± 0.46 ^b^** 10.8–12.2
Sweets and sugars	**0.075 ± 0.006 ^f^** 0.068–0.088	**0.099 ± 0.012 ^f^** 0.081–0.120	**0.33 ± 0.03 ^c^** 0.30–0.37	**0.418 ± 0.036 ^c^** 0.370–0.480	**0.14 ± 0.01 ^c^** 0.12–0.16	**0.219 ± 0.011 ^c^** 0.205–0.232	**1.39 ± 0.12 ^e^** 1.22–1.55	**1.60 ± 0.15 ^f^** 1.40–1.89	**31.8 ± 4.07 ^d^** 26.2–37.4	**16.2 ± 0.51 ^c^** 15.2–16.8	**8.83 ± 1.34 ^de^** 7.24–10.6	**6.36 ± 0.34 ^de^** 5.88–6.75
Water and beverages	**0.021 ± 0.002 ^abc^** 0.017–0.025	**0.029 ± 0.003 ^ad^** 0.025 - 0.035	**0.08 ± 0.014 ^ad^** 0.07–0.11	**0.193 ± 0.041 ^ab^** 0.130–0.250	**1.05 ± 0.06 ^f^** 0.97–1.14	**3.03 ± 0.23 ^f^** 2.72–3.31	**0.58 ± 0.04 ^d^** 0.52–0.63	**0.632 ± 0.087 ^de^** 0.480–0.780	**6.08 ± 1.03 ^a^** 4.69–7.46	**4.88 ± 0.64 ^a^** 4.19–5.96	**22.2 ± 5.23 ^g^** 12.4–28.3	**11.5 ± 2.90 ^b^** 8.00–16.1
Other products	**0.052 ± 0.005 ^d^** 0.047–0.061	**0.014 ± 0.002 ^bcd^** 0.011–0.017	**0.20 ± 0.02 ^abc^** 0.17–0.24	**0.079 ± 0.012 ^ab^** 0.060–0.100	**0.1 ± 0.007 ^bc^** 0.09–0.11	**0.035 ± 0.003 ^ab^** 0.032–0.039	**0.29 ± 0.05 ^ab^** 0.22–0.38	**0.149 ± 0.020 ^ab^** 0.120–0.180	**3.69 ± 0.50 ^ab^** 3.06–4.35	**3.56 ± 0.23 ^ab^** 3.12–3.85	**1.99 ± 0.15 ^ab^** 1.69–2.15	**1.16 ± 0.20 ^a^** 0.90–1.51
Total	**1.00 ± 0.04**	**1.01 ± 0.03**	**8.74 ± 0.37**	**10.2 ± 0.51**	**4.25 ± 0.19**	**5.66 ± 0.26**	**11.5 ± 0.55**	**15.9 ± 0.42**	**148 ± 7.00**	**177 ± 5.36**	**106 ± 8.00**	**119 ± 7.70**
EAR/AI	0.7	0.7	6.8	9.4	1.8	2.3	8	6	25	35	45	45

## Supplementary Materials

The following are available online at https://www.mdpi.com/2072-6643/12/11/3412/s1: Figure S1: Loadings of first two components of PCA, explaining together 78.86% of information in the dataset obtained for women (A), and 79.55% for men (B). Figure S2: Scores of first two principal components of PCA, explaining together 78.86% of information in the dataset obtained for women (A), and 79.55% for men (B).
