# Supplementary material for: Contribution of Major Groups of Food Products to the Daily Intake of Selected Elements—Results from Analytical Determinations Supported by Chemometric Analysis
Source: Nutrients. 2020 Nov 6;12(11):3412. doi: 10.3390/nu12113412 (PMC7694689; doi:10.3390/nu12113412)
Supplement: Supplementary file 1 [file nutrients-12-03412-s001.pdf]

SUPPLEMENTARY FILE

Contribution of major groups of food products to the daily intake of selected elements—results from analytical determinations supported by chemometric analysis

Wojciech Koch [1,*], Marcin Czop [2], Agnieszka Nawrocka [3] and Dariusz Wiącek [3]

[1] Chair and Department of Food and Nutrition, Medical University of Lublin, 4a Chodźki Str., 20-093 Lublin, Poland (W.K.)
[2] Department of Clinical Genetics, Medical University of Lublin, Radziwiłłowska 11 Str., 20-080 Lublin, Poland; marcin.czop@umlub.pl (M.C.)
[3] Institute of Agrophysics, Polish Academy of Sciences, Doświadczalna 4 Str., 20-290 Lublin, Poland; a.nawrocka@ipan.lublin.pl (A.N.) and d.wiacek@ipan.lublin.pl (D.W.)
* Correspondence: kochw@interia.pl; Tel.: +48-81-448-7143

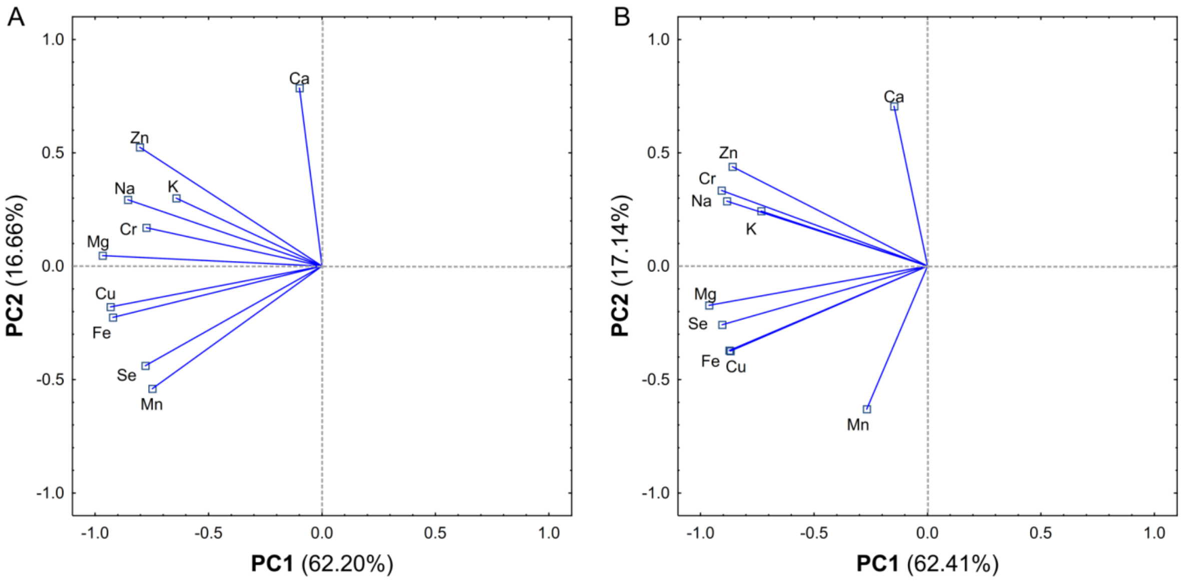

Figure S1. Loadings of first two components of PCA, explaining together 78.86% of information in the obtained dataset for women (A), and 79.55% for men (B).

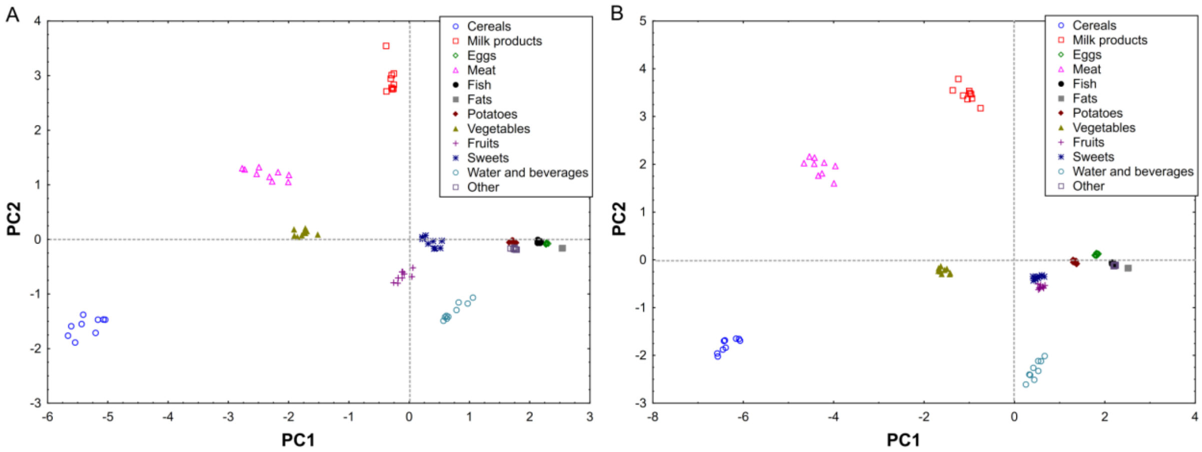

Figure S2. Scores of first two principal components of PCA, explaining together 78.86% of information in the obtained dataset for women (A), and 79.55% for men (B).
